# Intracortical facilitation and inhibition in human primary motor cortex during motor skill acquisition

**DOI:** 10.1007/s00221-022-06496-3

**Published:** 2022-10-29

**Authors:** Kelly Ho, John Cirillo, April Ren, Winston D. Byblow

**Affiliations:** 1 Movement Neuroscience Laboratory, Department of Exercise Sciences, The University of Auckland, Auckland, 1023 New Zealand; 2 Centre for Brain Research, The University of Auckland, Private Bag 92019, Auckland, 1023 New Zealand

**Keywords:** Skill acquisition, Visuomotor learning, Transcranial magnetic stimulation, Human primary motor cortex, Intracortical facilitation, Intracortical inhibition

## Abstract

The primary motor cortex (M1) is critical for movement execution, but its role in motor skill acquisition remains elusive. Here, we examine the role of M1 intracortical circuits during skill acquisition. Paired-pulse transcranial magnetic stimulation (TMS) paradigms of short-interval intracortical facilitation (SICF) and inhibition (SICI) were used to assess excitatory and inhibitory circuits, respectively. We hypothesised that intracortical facilitation and inhibition circuits in M1 would be modulated to support acquisition of a novel visuomotor skill. Twenty-two young, neurologically healthy adults trained with their nondominant hand on a skilled and non-skilled sequential visuomotor isometric finger abduction task. Electromyographic recordings were obtained from the nondominant first dorsal interosseous (FDI) muscle. Corticomotor excitability, SICF, and SICI were examined before, at the midway point, and after the 10-block motor training. SICI was assessed using adaptive threshold-hunting procedures. Task performance improved after the skilled, but not non-skilled, task training, which likely reflected the increase in movement speed during training. The amplitudes of late SICF peaks were modulated with skilled task training. There was no modulation of the early SICF peak, SICI, and corticomotor excitability with either task training. There was also no association between skill acquisition and SICF or SICI. The findings indicate that excitatory circuitries responsible for the generation of late SICF peaks, but not the early SICF peak, are modulated in motor skill acquisition for a sequential visuomotor isometric finger abduction task.

**Supplementary Information:**

The online version contains supplementary material available at 10.1007/s00221-022-06496-3.

## Introduction

The human nervous system demonstrates an impressive capacity for acquiring a plethora of skills. Motor skill learning describes the process by which movements are executed more efficiently as a result of practise (Willingham 1998). Skill acquisition, the initial phase of motor skill learning, is characterised by rapid improvements during training (Dayan and Cohen 2011). Repetitions of motor actions during skill acquisition involve sensorimotor mapping in the primary motor cortex (M1). Rapid functional and structural M1 reorganisation underlie sensorimotor mapping, presumably through long-term potentiation (LTP) mechanisms (Bütefisch et al. 2000; Wolpert et al. 2011). Although advances in non-invasive brain stimulation permit assessment of human M1 function, the neurophysiological processes that support motor skill acquisition are incompletely understood.

A single electric or magnetic pulse delivered over the M1 evokes a series of high-frequency (~ 660 Hz) discharge patterns in the descending corticospinal tract (Patton and Amassian 1954). The earliest volley may reflect direct activation of pyramidal neurons at their axons, whereas later volleys may reflect trans-synaptic activation of pyramidal neurons by intracortical circuits (Ziemann and Rothwell 2000). The mechanisms generating the indirect-waves (I-waves) remain unclear, but the most likely neural basis proposes physiologically distinct interneuronal networks projecting on different sites of the corticospinal tract (Ziemann 2020). Transcranial magnetic stimulation (TMS) can be used to investigate putative I-wave activity in the M1 from surface electromyographic (EMG) recordings. Short-interval intracortical facilitation (SICF) is a paired-pulse TMS paradigm that constitutes a suprathreshold first stimulus (S1) followed by a subthreshold or perithreshold second stimulus (S2). The SICF procedure gives rise to facilitated motor-evoked potential (MEP) amplitudes at distinct intervals separated by ~ 1.5 ms thought to reflect I-wave periodicity (Ziemann et al. 1998; Chen 2000; Hanajima et al. 2002).

Is motor skill acquisition mediated, at least in part, by the same circuits that generate I-waves in response to TMS? Previous studies indicate that I-wave recruitment may be involved in cortical plasticity and the acquisition of novel motor skills (Hamada et al. 2013; Sasaki et al. 2018). Distinct excitatory synaptic inputs to corticospinal neurons may contribute differently to various forms of motor learning. Specifically, the recruitment of late I-wave circuitries seemed to drive visuomotor learning (Hamada et al. 2014). In addition, movement preparation selectively modulates activity of late I-waves (Cattaneo et al. 2005; Cretu et al. 2020). I-wave generation is likely to be embedded with GABAergic processes (Di Lazzaro et al. 2012). Short-interval intracortical inhibition (SICI), a protocol where a subthreshold conditioning stimulus precedes a suprathreshold test stimulus by 1–6 ms, can probe GABA_A_-mediated inhibition within M1 (Kujirai et al. 1993). SICI enhances SICF related to late I-wave circuities, possibly through disinhibition (Wagle-Shukla et al. 2009). A reduction of SICI is also evident after a period of motor training, which is thought to promote cortical plasticity with training (Liepert et al. 1998; Bütefisch et al. 2000; Coxon et al. 2014; Mooney et al. 2019). Whether modulation of SICF and SICI is both associated with visuomotor skill acquisition is presently unknown.

The aim of the present study was to investigate the modulation of intracortical facilitation and inhibition during motor skill acquisition. We hypothesised that: (1) task performance would improve after skilled visuomotor training, but not after motor practise alone (non-skilled motor training); (2) SICF would increase and SICI would decrease after skilled visuomotor training, but not after motor practise alone (non-skilled motor training); and (3) there would be an association between the extent of skill acquisition and modulation in SICF and SICI.

## Methods

### Participants

Twenty-two neurologically healthy adults (9 males, mean age 23.4 ± 3.5, range 20–37) participated in the study. Handedness was assessed using the short version of the Edinburgh Handedness Inventory (Veale 2014) (20 right, laterality quotient 91.7 ± 15.5%; 2 left, laterality quotient − 100.0 ± 0.0%). All participants completed a TMS safety screening questionnaire and gave written informed consent. The study was approved by the University of Auckland Human Participants Ethics Committee (Ref. UAHPEC2583).

### Experimental design

**Fig. 1:**
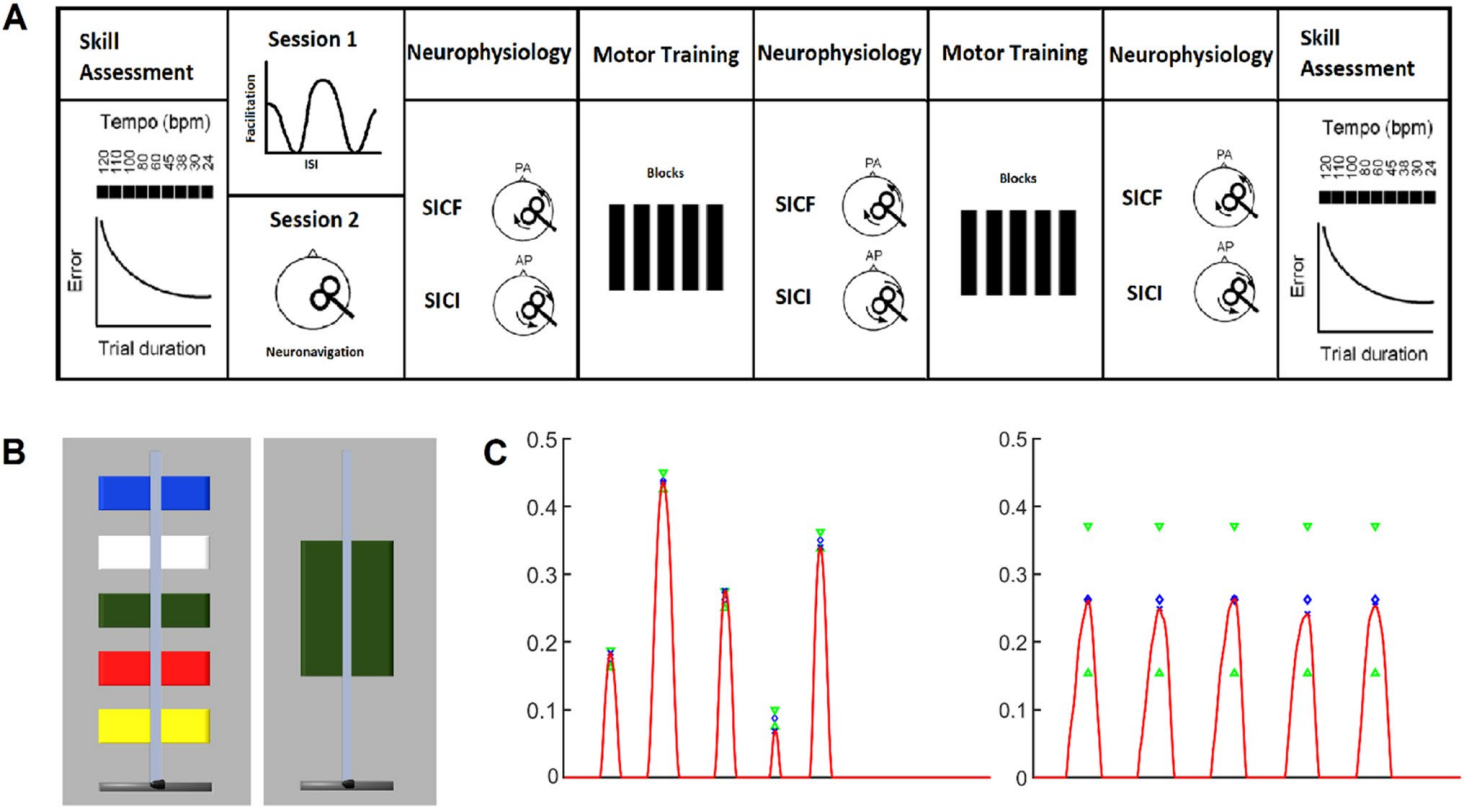
Experimental design. **A** Each participant underwent a skilled and non-skilled acquisition session in a randomised order. Skill was quantified at the beginning and end of each session. Transcranial magnetic stimulation (TMS) with posterior–anterior (PA) current was used to measure short-interval intracortical facilitation (SICF) and corticomotor excitability, whereas anterior–posterior (AP) current was used to measure short-interval intracortical inhibition (SICI). Neurophysiological measures were probed before, mid, and after each 10-block training session. **B** The on-screen display for the skilled sequential isometric force task (left) and non-skilled unitary isometric force task (right). Each trial required index finger abduction against a force transducer. In the skilled task, participants reached the targets in the order red–blue–green–yellow–white. In the non-skilled task, participants were required to reach the green target five times. **C** A representative force peak from a single participant performing the five-sequence target sequence (left) and the unitary target sequence (right) with the nondominant index finger against the force transducer

Participants completed two sessions in total, performing a skilled or non-skilled motor task, in a pseudorandomised order. At the start of each session, a speed–accuracy function (SAF) was obtained for each participant as a measure of skill. The SAF involved a sequential isometric force task and a unitary isometric force task for the skill acquisition session and the non-skilled session, respectively. Participants were then trained (10 blocks of 10 trials) on the respective task, and the SAF was reassessed at the end of motor training. Intracortical facilitatory and inhibitory circuits were assessed at rest using TMS at three time points relative to motor training. In the first session, SICF was investigated over a range of interstimulus intervals (ISIs) that coincided with the three peaks of facilitation. The ISIs where facilitation was maximal for each peak (SICF_Peaks_) were selected for each participant and used in both sessions. At each of the time points before, at the midway point, and after motor training, SICF_Peaks_ and SICI were assessed (Fig. 1A). Sessions were completed at the same time of day for each participant and separated by at least a week (mean 11 days, range 7–36 days). For each participant, the same stimulation site was used across sessions.

### Task

Participants sat in front of a computer screen with their elbow and forearm positioned on a table directly in front of their chair. The nondominant hand was placed in the apparatus on the table, such that the distal interphalangeal joint of the index finger rested against a force transducer (NI, Austin, Texas, USA), while the thumb and middle fingers were restricted (Coxon et al. 2014; Mooney et al. 2019). The nondominant hand was used to maximise the room for improvement and avoid a ceiling effect in task performance (Ridding and Flavel 2006). Index finger abduction against the transducer displaced an on-screen cursor vertically (Fig. 1B).

In the skilled task, participants performed a sequential isometric force task, where the goal was to produce five individual force peaks by moving the cursor to five fixed targets on the screen in a specific colour sequence (red–blue–green–yellow–white) and returning to the home position between each colour (Reis et al. 2009; Coxon et al. 2014; Mooney et al. 2019). In the non-skilled task, participants performed a unitary isometric force task, where the goal was to produce five individual force peaks by moving the cursor repetitively into one large (green) target on the screen and returning to the home position between each force peak (Fig. 1C). Therefore, the two tasks were matched for the average required force production, assuming peak force occurred at the centre of each target. The furthest target was set to 45% of the participant's maximum voluntary abduction strength, determined from the highest of three brief maximal isometric index finger abduction trials performed in the first session. The other targets in decreasing order were set to 35%, 26%, 18%, and 9% of maximum voluntary contraction, respectively. Logarithmic transformation was applied to the relationship between applied force and cursor movement (Reis et al. 2009; Coxon et al. 2014; Stavrinos and Coxon 2017; Mooney et al. 2019; Cirillo et al. 2020).

### Skill assessment

Skill was determined by measuring error at fixed execution speeds to compute the SAF (Reis et al. 2009). During skill assessments, auditory metronome beats were presented to participants, who were instructed to move the cursor to each of the targets in time with the beat. In the skilled session, participants aimed to place the cursor at the centre of the target on the beat as accurately as possible; in the non-skilled session, participants moved the cursor anywhere within the bounds of the large target on the beat. The assessment comprised completing a single block of three trials at nine different tempos 120/110/100/80/60/45/38/30/24 bpm, or 2/1.83/1.67/1.33/1/0.75/0.63/0.5/0.4 Hz, which corresponded to approximate trial durations of 2.5/2.75/3/3.75/5/6.65/7.9/10/12.5 s, respectively. Block order was randomised and a 30 s rest period occurred between blocks.

### Training

Participants completed 10 blocks of 10 trials (100 trials in total) halved in 5-block stages with a 1 min rest period between each block. In the skilled task, participants were instructed to complete the training trials at a self-selected pace, with the aim of completing each trial as quickly and accurately as possible (Reis et al. 2009). Participants received visual feedback of their training performance, along with performance-dependent messages: (1) Well done! Your skill has increased compared with the previous block, or (2) Try harder! Your skill has decreased compared with the previous block. In the non-skilled task, participants were instructed to complete the training trials at an approximate rate of 1 Hz with the aim to maintain performance. Participants received visual feedback of their mean training performance and were verbally encouraged to maintain training performance throughout the blocks. Neurophysiological measures were obtained in the relaxed FDI muscle before, at the midway point of training between the first and second stages (hereafter referred to as ‘mid’ training), and after motor training.

### Electromyography

Participants were seated comfortably in a chair with back support and arms supported by a pillow placed in their lap. Surface EMG was recorded from the nondominant first dorsal interosseous (FDI) muscle using adhesive 20 mm pre-gelled recording electrodes (Conmed Corp., Utica, New York, USA) arranged in a belly–tendon montage. A ground electrode (Conmed Corp., Utica, New York, USA) was placed on the dorsum of the same hand. EMG signals were amplified (× 1000) and band-pass filtered (10–1000 Hz) using an AMT-8 amplifier (Bortec Biomedical, Calgary, Canada), and sampled at 2000 Hz using a micro1401 mkII data acquisition board (Cambridge Electronic Design Ltd., Cambridge, UK). Data were acquired using Signal Software (version 6.05; Cambridge Electronic Design Ltd., Cambridge, UK) and saved for offline data analysis.

### Transcranial magnetic stimulation

TMS was always performed in the relaxed FDI after 5 min of rest following training blocks to prevent fatigue. Single- and paired-pulse TMS were delivered using a figure-of-eight coil (70 mm wing diameter) through a Magstim BiStim^2^ with two 200^2^ units connected via a connecting module (MagStim, Whitland, Wales, UK). The coil was oriented ~ 45° to the midline and delivered monophasic current. The optimal site to elicit consistent MEPs in the nondominant FDI with posterior–anterior (PA) current was marked on the scalp. The same scalp position was used for anterior–posterior (AP) current.

The site of stimulation in the first session was used for the second session. Using a frameless stereotaxic neuronavigation system (Brainsight TMS Frameless Navigation System; Rogue Research Inc., Montreal, Quebec, Canada), participants were spatially registered to a 3D-constructed MNI152 brain template. The TMS coil and location were recorded and used for image-guided TMS positioning in the second session. This site was marked, confirmed, and used as the optimal site to elicit MEPs.

### Motor thresholds

Motor thresholds were determined over 16 trials by adaptive threshold-hunting using maximum-likelihood parameter estimation by sequential testing (PEST) without a priori information (Awiszus 2003). For resting motor threshold (RMT), a PA-induced current was delivered and a trial was deemed successful if the stimulus intensity elicited an MEP of at least 50 µV in amplitude (Rossini et al. 2015). For active motor threshold (AMT), an AP-induced current was delivered and a trial was deemed successful if the stimulus intensity elicited an MEP of at least 200 µV in amplitude, while the FDI was pre-activated to approximately 10% of the participant’s perceived maximum voluntary contraction. RMT was determined using an automated adaptive threshold-hunting paradigm in MATLAB (R2018b; MathWorks, Natick MA, USA) that adjusted TMS output intensity as required according to the PEST procedure (Calvert et al. 2020). Adaptive threshold-hunting for AMT was performed using freeware (TMS Motor Threshold Assessment Tool; MTAT 2.0, F. Awiszus and J. Borckardt), which was more amenable to detecting thresholds in the presence of voluntary EMG.

### Intracortical facilitation and inhibition

For SICF, TMS was delivered in a PA-induced current direction. At the start of the first session, SICF was investigated over a range of ISIs. A suprathreshold stimulus S1 with intensity set to elicit MEP amplitude of 0.8–1.5 mV, determined by automated adaptive threshold-hunting, was followed by a subthreshold S2 set to 90% RMT (Ziemann et al. 1998; Hanajima et al. 2002). Ten ISIs in each of the facilitatory windows 1.1–1.5 ms, 2.3–2.7 ms, and 3.9–4.5 ms were tested in 0.2 ms intervals (Chen and Garg 2000). Ten stimuli for each ISI, and 20 for the non-conditioned state (S1 given alone), were collected in a randomised order. From the SICF curve, the largest mean peak-to-peak MEP amplitude in each facilitatory window was selected as ISI_Peak 1_, ISI_Peak 2_, and ISI_Peak 3_, respectively, for each participant. SICF_Peak_ blocks were composed of four states: 12 non-conditioned stimuli (S1 alone) and 12 stimuli at each of the three individualised peak ISIs, with the order of presentation randomised throughout the trials (48 trials in total). Stimulation intensities were consistent with those described above for the SICF curve.

For SICI, TMS was delivered in an AP-induced current direction and was quantified using automated adaptive threshold-hunting. A non-conditioned threshold-hunting target (THT) was obtained whereby the MEP amplitude criterion for a successful trial was 200 µV. A subthreshold conditioning stimulus set to 90% AMT was delivered 3 ms before the test stimulus (Cirillo et al. 2018). In the presence of the conditioning stimulus, the test stimulus intensity was adjusted to reach the adaptive THT of 200 µV, also called the adjusted THT (THT_adj_).

## Data analysis

### Skill

For each trial in the skill assessment, an error value was calculated as the sum of the Euclidean difference between the centre of each target and the five respective force peaks (Coxon et al. 2014). To quantify skill for each participant at each time point, we used the following function (Reis et al. 2009):

$$\text{Skill parameter}= \frac{1-\mathrm{error}}{\mathrm{error}\times \mathrm{ln}{(\text{trial duration})}^{\mathrm{b}}},$$

where error is the mean error for the respective trial duration (s) and *b* is the dimension-free parameter. For SAF, the Curve Fitting Tool in MATLAB was used to optimise the fit of the skill function (Mathworks, MA). The skill parameter was determined from the optimised curve for each participant (skilled task pre-training R-squared = 0.74 ± 0.12). The skill measure was the logarithm of the skill parameter, which served to homogenise variance (Reis et al. 2009; Stavrinos and Coxon 2017).

The skill parameter for the training trials (hereby referred to as training performance) was quantified using the above equation, with the exception that trial duration (s) was derived from the onset of the first force peak to the last force peak (i.e., movement time). Mean performance for the block of trials was presented as visual feedback during rest periods. The *b* value was set to that calculated from the pre-training SAF for each participant. Mean training performance, movement error, and movement time were extracted for each block.

### Neurophysiology

For procedures involving automated adaptive threshold-hunting, a trial was immediately rejected and repeated if root mean square (RMS) of background EMG activity over a 50 ms pre-trigger window exceeded 10 μV.

**Fig. 2:**
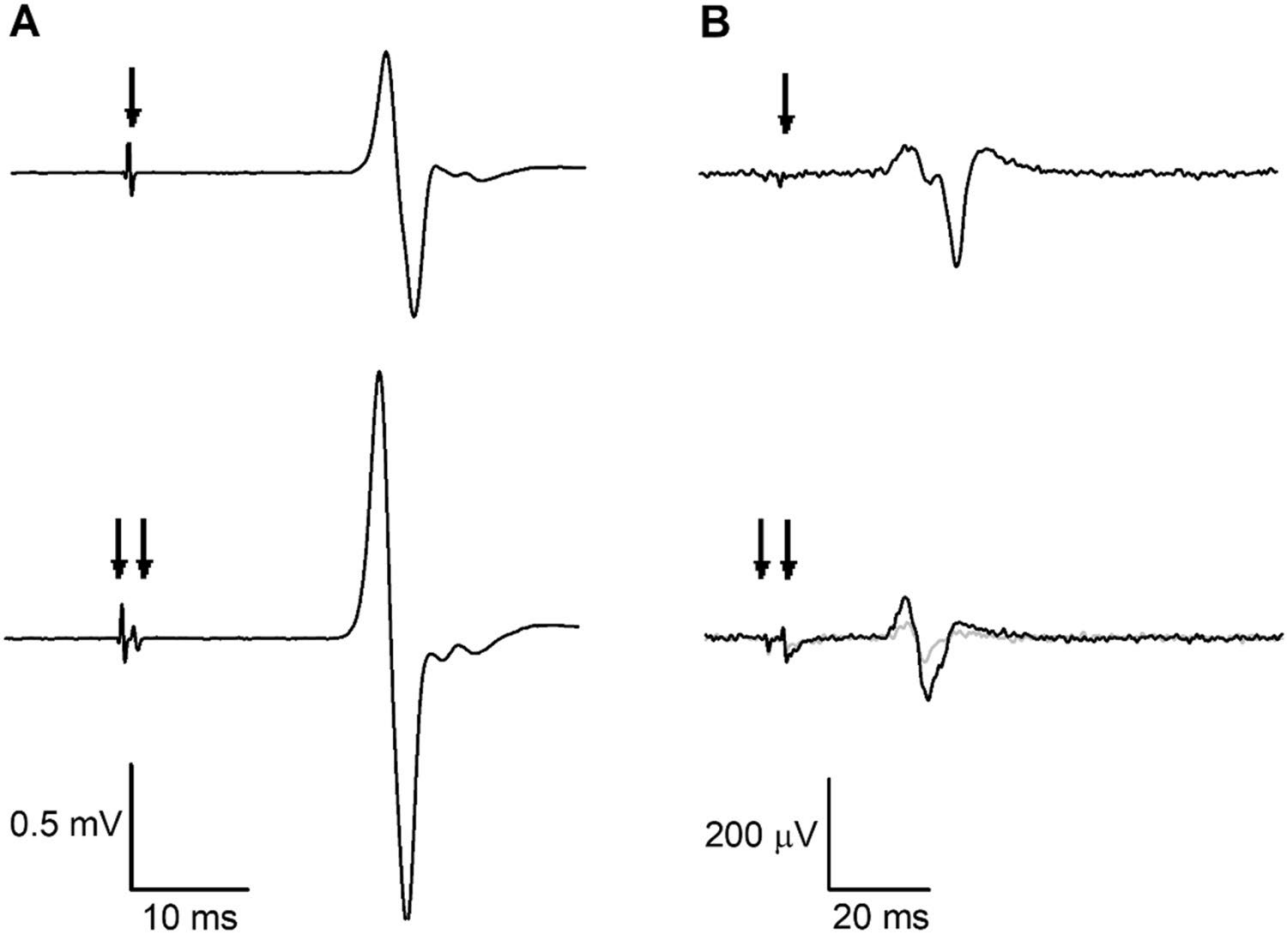
Representative electromyographic (EMG) traces showing motor-evoked potentials (MEPs) from the nondominant first dorsal interosseous (FDI) muscle of a single participant. Arrows indicate transcranial magnetic stimulation (TMS) artefacts. **A** Short-interval intracortical facilitation (SICF). Top trace: non-conditioned MEP with suprathreshold first stimulus (S1) given alone; bottom trace: conditioned MEP with S1 followed by subthreshold second stimulus (S2). **B** Short-interval intracortical inhibition (SICI). Top trace: non-conditioned MEP with test stimulus given alone (threshold-hunting target, THT; 200 µV); bottom trace: conditioned MEP, where the grey trace represents unadjusted THT and the black trace represents adjusted THT in the presence of the conditioning stimulus

For SICF, trials where RMS background EMG activity over a 50 ms pre-trigger window exceeded 10 µV were discarded (mean 0.2%, range 0–1.7%). Outliers of peak-to-peak MEP amplitudes were excluded if

$$x < {\text{Q1}} - 1.5 \times {\text{IQR}};\,{\text{or}}\,,\,x > {\text{Q3}} + 1.5 \times {\text{IQR}},$$

where Q1 is the lower quartile, Q3 is the upper quartile, and IQR is the interquartile range (Wilcox 2010). A mean of 4.4% (range of 2.1–7.6%) of trials was excluded from each participant due to outliers. Mean peak-to-peak MEP amplitudes were averaged from ≥ 8 trials for each condition. Facilitation was expressed as a relative percentage of the mean non-conditioned MEP amplitude (Fig. 2)

$$\text{Facilitation}= \frac{\text{Conditioned}}{\text{Non Conditioned}}\times 100,$$

where values greater than 100 indicated facilitation and values below 100 indicated inhibition. Three participants had small mean non-conditioned MEP amplitudes that were classified as outliers and excluded from analysis for each task.

SICI was expressed as the percentage threshold change of the adjusted THT (THT_adj_) relative to the non-conditioned THT (Fig. 2)

$$\text{Threshold change}= \frac{\mathrm{{THT}_{adj}}-\mathrm{THT}}{\mathrm{THT}}\times 100,$$

where positive values indicated more inhibition and negative values indicated facilitation.

### Statistical analysis

Normality was assessed using the Shapiro–Wilk’s test and homoscedasticity of variance using Levene’s test of equality and Mauchly’s test of sphericity. Non-normal data (Facilitation, 1 mV) were log-transformed, which corrected their normality. Pre-training neurophysiological variables (RMT, AMT, MEP_1mV_, and THT) between the two tasks were analysed using paired t tests. One-sample t tests were performed on pre-training SICF (hypothesised mean = 100) and SICI (hypothesised mean = 0) to confirm significant facilitation and inhibition at baseline for both tasks. An order effect was investigated with two sample *t* tests between the baseline skill of the skilled task with and without prior exposure to the non-skilled task. Change in skill of the SAF for each task was analysed using a linear mixed-effects model with fixed effects of TASK (skilled, non-skilled), TIME (pre-training, post-training), TASK × TIME interaction, and random effects of PARTICIPANT. Change in training performance, movement time, and movement error across the two training stages (first: blocks 1–5; second: blocks 6–10) were analysed using linear mixed-effects models with fixed effects of TASK (skilled, non-skilled), BLOCK (Block 1, Block 5, Block 6, Block 10), TASK × BLOCK interaction, and random effects of PARTICIPANT. SICF at each facilitatory peak (Peak 1, Peak 2, Peak 3) was analysed using a linear mixed-effects model with fixed effects of TASK (skilled, non-skilled), TIME (before, mid, and after), TASK × TIME interaction, and random effects of PARTICIPANT. For SICI, THT, and non-conditioned MEP_1mV_ amplitude, data were analysed using linear mixed-effects models with fixed effects of TASK (skilled, non-skilled), TIME (before, mid, after), TASK × TIME interaction, and random effects of PARTICIPANT. For all linear mixed-effects models, random slopes were included for the factor Task. Pearson correlation analyses (two-tailed) were used to investigate the relationship between the magnitude of skill acquisition and training performance, and between the magnitude of skill acquisition and modulation of SICF and SICI. The significance level was set at *p* < 0.05. Post hoc Šidák tests with a significance threshold of *p* = 0.05 were used to test for significant comparisons. Group data are presented as mean ± SD in the text.

## Results

Eighteen participants completed the study with no adverse effects from the procedures. Four participants did not complete the study due to high motor thresholds with AP current which precluded the AMT/THT/SICI procedure.

### Baseline measures

Average baseline neurophysiological and behavioural measurements determined for each session are shown in Table 1. RMT, AMT, MEP_1mV_, and the non-conditioned MEP amplitude did not differ between skilled and non-skilled experimental sessions. In each experimental session, SICF was greater than 100 and inhibition was greater than 0, indicating that the protocols successfully elicited facilitation and inhibition. Baseline performance of the non-skilled was higher than the skilled task. The pre-training *b* value was 1.40 ± 0.65 for the skilled task and 0.51 ± 0.54 for the non-skilled task.

**Table 1:** Baseline neurophysiological and behavioural measures

	Skilled	One-sample *t* test *p* value	Non-skilled	One-sample *t* test *p* value	Paired *t* test *p* value
PA stimulation					
RMT (%MSO)	52.56 (± 8.89)	–	53.17 (± 9.52)	–	0.520
MEP_1 mV_ (%MSO)	66.28 (± 10.86)	–	66.72 (± 12.70)	–	0.962
NC amplitude (mV)	1.08 (± 0.58)	–	1.35 (± 0.78)	–	0.307
SICF (% NC)					
Peak 1	214 (± 123)	** < 0.001**	145 (± 39)	** < 0.001**	0.204
Peak 2	209 (± 178)	** < 0.001**	126 (± 31)	**0.003**	0.097
Peak 3	136 (± 91)	0.117	102 (± 26)	0.587	0.188
AP stimulation					
AMT (%MSO)	60.24 (± 11.26)	–	58.88 (± 10.60)	–	0.284
SICI (% threshold change)					
THT (%MSO)	71.53 (± 12.74)	–	73.82 (± 13.98)	–	0.970
THT_adj_ (%MSO)	79.00 (± 11.61)	–	81.38 (± 10.24)	–	0.441
Inhibition (% ΔTHT)	20.06 (± 12.46)	** < 0.001**	19.35 (± 7.96)	** < 0.001**	0.837
Behavioural					
Skill parameter - SAF	0.87 (± 0.68)	–	1.50 (± 0.29)	–	** < 0.001**

Data are presented as mean (± standard deviation)

*AMT* active motor threshold, *AP* anterior–posterior current, *MEP*_*1mV*_ stimulus intensity to elicit MEP of 1 mV amplitude, *MSO* maximum stimulator output, *NC* non-conditioned stimulus, *PA* posterior-anterior current, *RMT* resting motor threshold, *SICF* short-interval intracortical facilitation, *SICI* short-interval intracortical inhibition, *THT* threshold-hunting target, *THT*_*adj*._ adjusted threshold-hunting target, *SAF* speed-accuracy function

Bold values indicate *p* < 0.05

### Skill

**Fig. 3:**
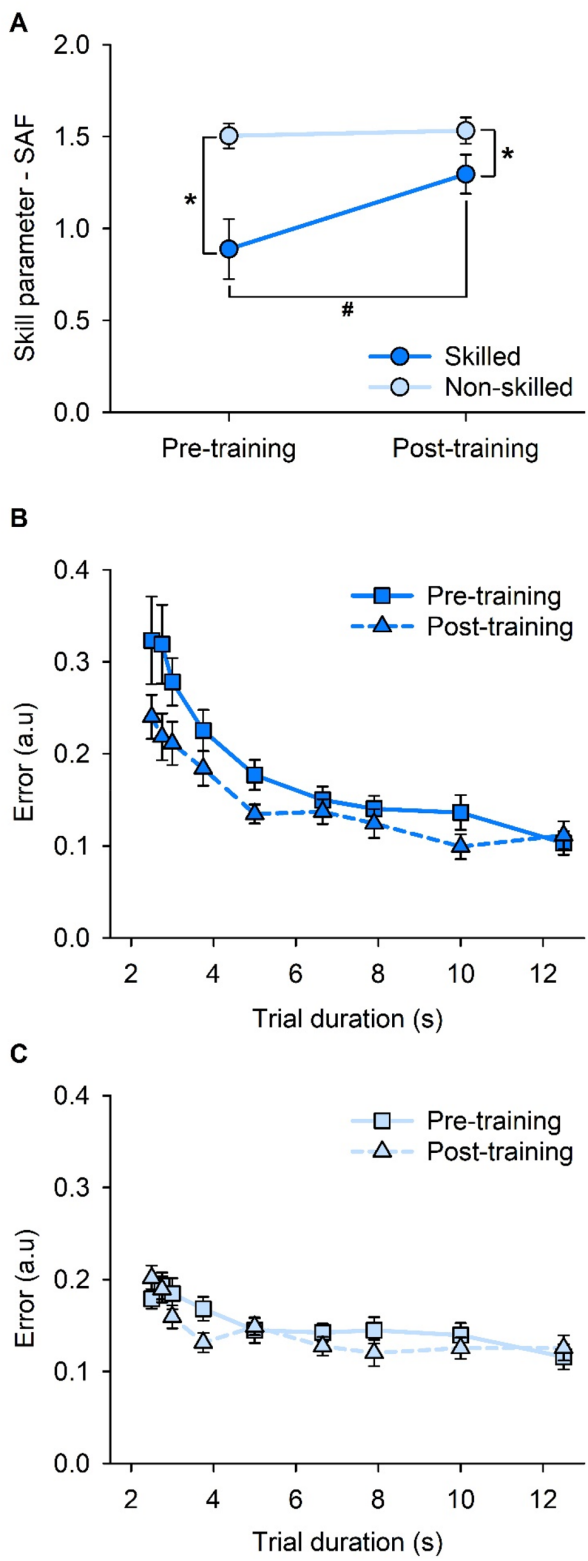
Speed–accuracy function (SAF). **A** Skill values (i.e., skill parameter—SAF) pre- and post-training on the skilled and non-skilled task. **B** SAF data obtained pre- and post-training on the skilled task. **C** SAF data obtained pre- and post-training on the non-skilled task. Error bars represent standard error of the mean. *p < 0.05 between skilled and non-skilled tasks. ^#^*p* < 0.05 between pre-training and post-training skill

Results of the linear mixed-effects model for change in skill parameter of the SAFs are shown in Fig. 3A, with SAFs for the skilled and non-skilled tasks shown in Figs. 3B, C respectively. There were main effects of TASK (*F*_1,51_ = 32.872; *p* < 0.001) and TIME (*F*_1,51_ = 8.937; *p* = 0.004), and an interaction of TASK × TIME (*F*_1,51_ = 6.804; *p* = 0.012). Post hoc pairwise comparison analyses revealed an improvement in skill after skilled training (pre-training = 0.87 ± 0.68, post-training = 1.30 ± 0.45; *p* = 0.003). There was no improvement in skill after non-skilled training (pre-training = 1.50 ± 0.29, post-training = 1.53 ± 0.30; *p* = 0.715).

Two-sample *t* tests indicate no order effect of sessions. Previous exposure to the skilled task did not increase mean non-skilled baseline performance (prior exposure = 0.72 ± 0.07, no prior exposure = 1.10 ± 0.03; *t* = − 1.944; *p* = 0.399). Similarly, previous exposure to the non-skilled task did not increase mean skilled baseline performance (prior exposure = 1.56 ± 0.46, no prior exposure = 1.45 ± 0.23; *t* = 0.771; *p* = 0.661).

### Training

**Fig. 4:**
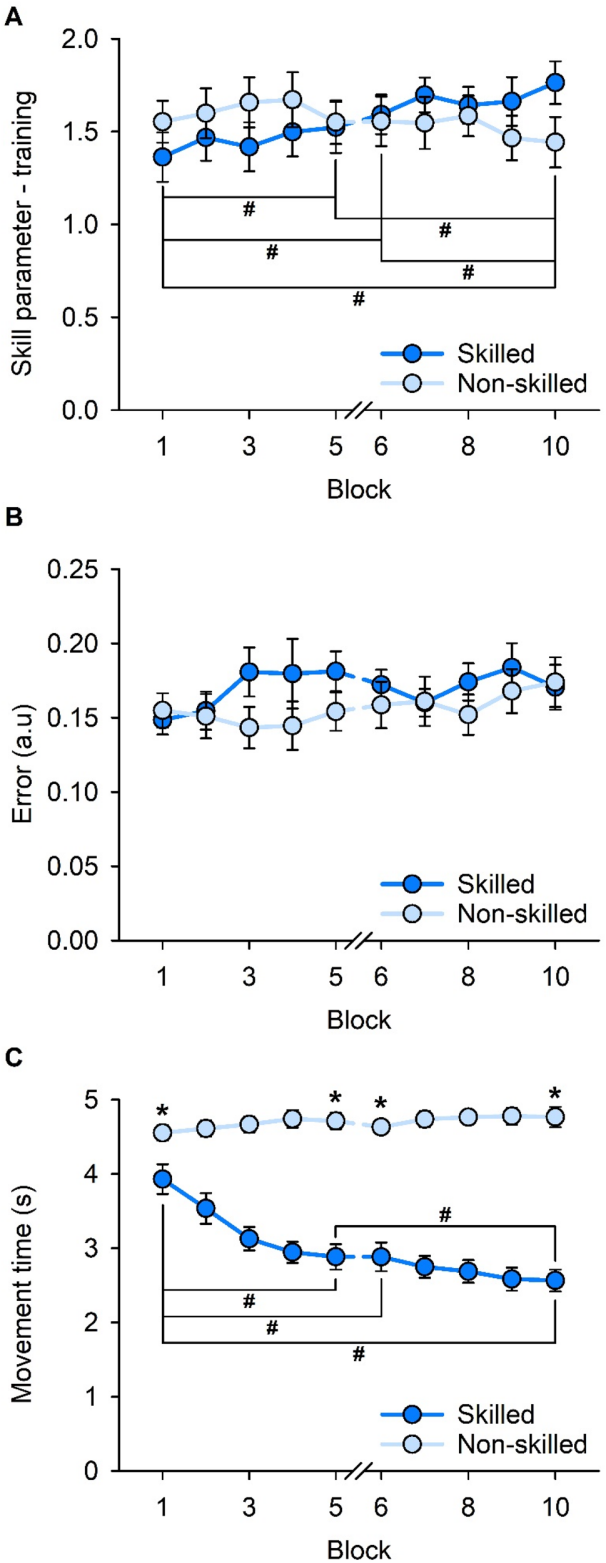
Training performance across the 10 blocks of skilled and non-skilled task training. **A** Overall training performance (i.e., skill parameter-training) across training blocks. **B** Mean movement error across training blocks. **C** Mean movement time across training blocks. Error bars represent standard error of the mean. *p < 0.05 between the two tasks. ^#^*p* < 0.05 between training blocks

Results of the linear mixed-effects model for training performance (i.e., skill parameter—training) are shown in Fig. 4A. Training performance was further decomposed into movement error and movement time, and results of the linear mixed-effects model for each component are shown in Fig. 4B, C, respectively.

For training performance, there was no main effect of TASK (*F*_1,17_ = 0.077; *p* = 0.785), a main effect of BLOCK (*F*_3,102_ = 2.980; *p* = 0.035), and a TASK × BLOCK interaction (*F*_3,102_ = 8.588; *p* < 0.001). Post hoc pairwise comparison analyses revealed an increase in mean performance in the skilled task from the beginning of the first training stage (Block 1 = 1.36 ± 0.56) to the end of the first training stage (Block 5 = 1.52 ± 0.58; *p* = 0.043), the beginning of the second training stage (Block 6 = 1.59 ± 0.45; *p* = 0.014), and end of the second training stage (Block 10 = 1.76 ± 0.49; *p* < 0.001). There was also an increase in mean performance between Block 10 (end of the second training stage) and Block 5 (end of the first training stage; *p* = 0.001) and Block 6 (beginning of the second training stage; *p* = 0.011), but not between Blocks 5 and 6 (*p* = 0.515). There were no changes to the mean performance in the non-skilled task (all *p* > 0.137), and mean training performance was not different between the skilled and non-skilled tasks at the beginning and end of each training stage (all *p* > 0.061).

For movement error, there was no main effect of TASK (*F*_1,17_ = 0.334; *p* = 0.571), BLOCK (*F*_3,102_ = 2.170; *p* = 0.096), and no TASK × BLOCK interaction (*F*_3,102_ = 1.699; *p* = 0.172).

For movement time, there were main effects of TASK (*F*_1,17_ = 71.841; *p* < 0.001), BLOCK (*F*_3,102_ = 15.774; *p* < 0.001), and an interaction of TASK × BLOCK (*F*_3,102_ = 27.806; *p* < 0.001). Post hoc pairwise comparison analyses revealed a reduction in mean movement time for the skilled task between the first training stage (Block 1 = 3.93 ± 0.85) and the end of the first training stage (Block 5 = 2.88 ± 0.73; *p* < 0.001), the beginning of the second training stage (Block 6 = 2.88 ± 0.82; *p* < 0.001), and end of the second training stage (Block 10 = 2.57 ± 0.62; *p* < 0.001). There was also a reduction in movement time between Block 5 (end of the first training stage) and Block 10 (end of the second training stage; *p* = 0.003). All other comparisons for movement time in the skilled task were not different (all *p* > 0.198). For the non-skilled task, there were no changes in mean movement time (all *p* > 0.132) and mean movement time was shorter for the skilled task compared with non-skilled at the beginning and end of each training stage (all *p* < 0.016).

### Correlation between skill acquisition and training performance

No correlations between the magnitude of skill acquisition and the magnitude of training improvements were observed for any comparison (all *p* > 0.231, after corrections; Supplemental Fig. 1).

### Short-interval intracortical facilitation

**Fig. 5:**
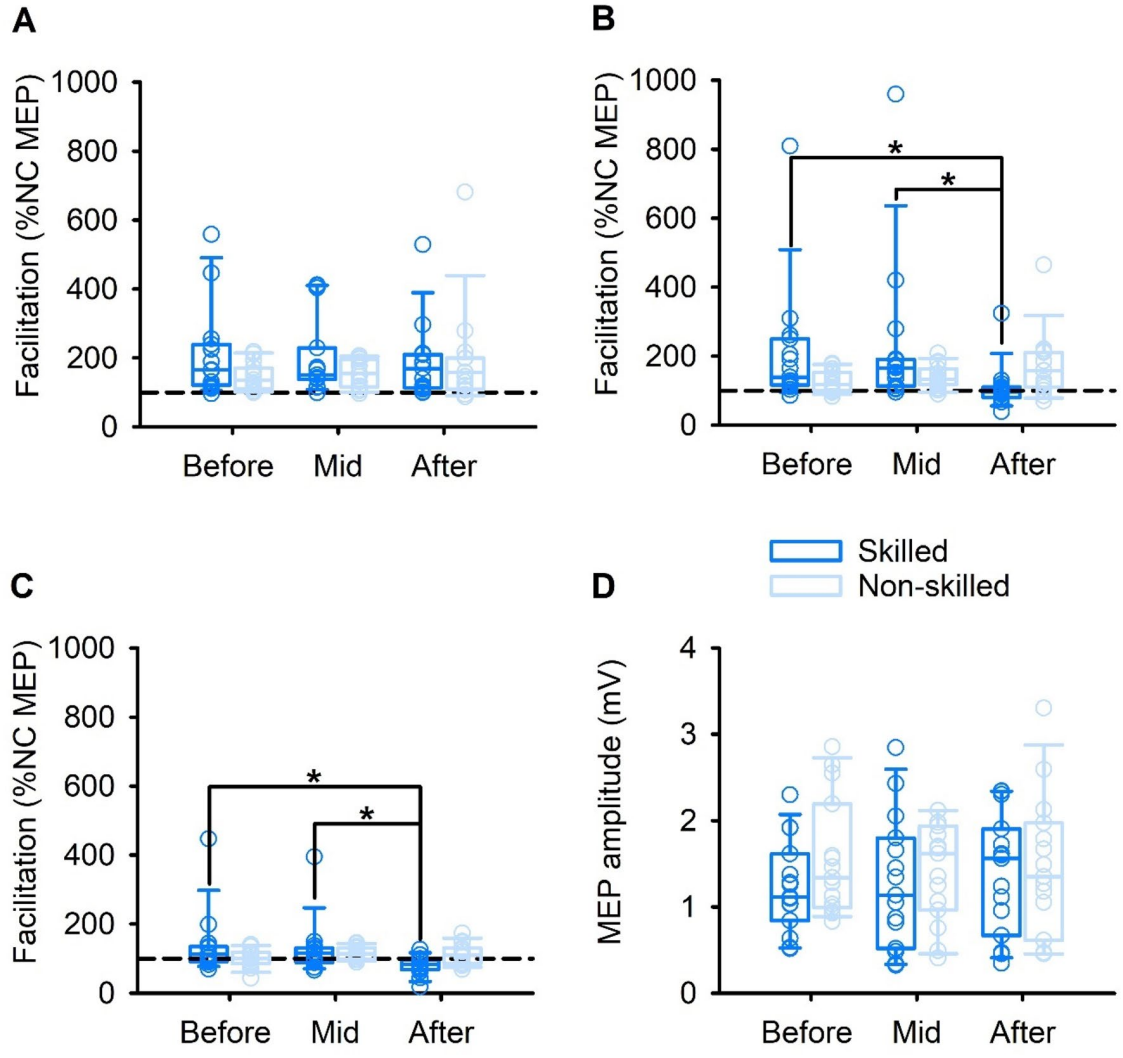
Short-interval intracortical facilitation (SICF) and corticomotor excitability before, mid, and after skilled and non-skilled task training. **A** SICF for Peak 1. **B** SICF for Peak 2. **C** SICF for Peak 3. **D** Motor-evoked potential (MEP) amplitude. Boxes, 25th and 75th percentiles; whiskers, 10th and 90th percentiles. *p < 0.05

Results of the linear mixed-effects model for each SICF peak before, mid, and after motor training for each of the skilled and non-skilled motor tasks are shown in Fig. 5.

For Peak 1, there were no main effects of TASK (*F*_1,13.1_ = 1.575; *p* = 0.231) or TIME (*F*_2,52.3_ = 0.577; *p* = 0.565), and there was no TASK × TIME interaction (*F*_2,52.3_ = 0.820; *p* = 0.446).

For Peak 2, there was no main effect of TASK (*F*_1,10.5_ = 0.185; *p* = 0.676), a main effect of TIME (*F*_2,48.6_ = 4.450; *p* = 0.017), and a TASK × TIME interaction (*F*_2,48.6_ = 7.850; *p* = 0.001). Post hoc pairwise comparison analyses revealed a reduction in SICF for the skilled task after training (108.86 ± 16.36%), compared to before (208.92 ± 46.05%; *p* = 0.014) and mid-training (226.84 ± 54.46%; *p* = 0.015). There were no changes in SICF after non-skilled training (*p* = 0.572).

For Peak 3, there was no main effect of TASK (*F*_1,72.4_ = 2.902; *p* = 0.093), a main effect of TIME (*F*_2,66.3_ = 3.745; *p* = 0.029), and a TASK × TIME interaction (*F*_2,66.3_ = 3.542; *p* = 0.035). Post hoc pairwise comparison analyses revealed a reduction in SICF for the skilled task after training (80.24 ± 6.95%), compared to before (136.43 ± 23.52%; *p* = 0.022) and mid-training (129.04 ± 19.95%; *p* = 0.041). There were no changes in SICF after non-skilled training (*p* = 0.370).

### Corticomotor excitability in PA-sensitive circuits

Results of the linear mixed-effects model for corticomotor excitability before, mid, and after motor training for each of the skilled and non-skilled tasks are shown in Fig. 5D. There were no effects of TASK (*F*_1,17_ = 0.135; *p* = 0.717) or TIME (*F*_2,68_ = 0.649; *p* = 0.526), and no TASK × TIME interaction (*F*_2,68_ = 0.567; *p* = 0.570).

### Correlation between facilitation and skill

No correlations between the magnitude of skill acquisition and the magnitude of SICF were observed for any comparison (all *p* > 0.370, after corrections; Supplemental Figure 2).

### Inhibition

Eight participants were included in the SICI analysis. The other participants were excluded due to high thresholds and the conditioned MEP not reaching THT in the baseline condition. Results of the linear mixed-effects analysis for SICI before, mid, and after motor training for each of the skilled and non-skilled motor tasks are shown in Supplemental Figure 3. There were no effects of TASK (*F*_1,7_ = 0.243; *p* = 0.637) or TIME (*F*_2,28_ = 1.908; *p* = 0.167), and no TASK × TIME interaction (*F*_2,28_ = 0.842; *p* = 0.442). For the THT, an ancillary measure of corticomotor excitability in AP-sensitive circuits, results of the linear mixed-effects model showed no effects of TASK (*F*_1,12_ = 0.237; *p* = 0.635) or TIME (*F*_2,48_ = 0.078; *p* = 0.925), and no TASK × TIME interaction (*F*_2,48_ = 0.511; *p* = 0.603).

### Correlation between inhibition and skill

No correlations between the magnitude of skill acquisition and the magnitude of SICI were observed for any comparisons (all *p* > 0.119, after corrections; Supplemental Figure 4).

## Discussion

The present study investigated the modulation of intracortical facilitation and inhibition during motor skill acquisition. The SICF and SICI protocols successfully elicited facilitation and inhibition, respectively (Table 1). In support of the first hypothesis, task performance improved after skilled, but not non-skilled, task training. In opposition to the second hypothesis, there was a reduction in later SICF peaks with skilled task training but not with non-skilled task training, with no modulation of SICI or corticomotor excitability with either task. Finally, there were no associations between the magnitude of skill acquisition and training performance, and no associations between the magnitude of skill acquisition and the magnitude of SICF or SICI. Together, these findings indicate that late SICF peak activity may be modulated by motor skill acquisition of a sequential visuomotor isometric finger abduction task in the absence of GABA_A_-mediated inhibition and corticomotor excitability modulation.

### Skill acquisition

M1 is a critical brain region for acquiring a new motor skill in skilled motor behaviours. Skill increased for skilled task training only (Reis et al. 2009; Mooney et al. 2019). The present task provides a skill measure based on movement accuracy at fixed speeds to account for the speed–accuracy trade-off (Reis et al. 2009). While previous studies have reported improvements in speed or accuracy (Karni et al. 1998; Korman et al. 2003), these measures alone may have simply reflected a speed-accuracy trade-off (Fitts 1954). The improvement from baseline performance in the skilled task reflects acquisition of novel movement dynamics and kinematics. Sequence learning and precision control are features of the skilled training task that implicate greater engagement and effort compared to the non-skilled training task. The increase in skill after training may reflect improved sensorimotor mapping (Wolpert et al. 2011). Furthermore, the improvement in task performance likely reflects an increase in movement speed rather than a reduction in movement error. In contrast to the skilled task, there was no improvement in skill after repeated practise of the non-skilled task. While minimal demands on precision or a ceiling effect in performance may contribute to the lack of improvement in the non-skilled task, and higher baseline performance compared with the skilled task, the instructions provided helped eliminate task performance improvements while controlling for motor output. The absence of improvement at the end of the non-skilled training reflects that motor practise alone did not induce skill acquisition as no task dynamics and kinematics were learnt. The formation of novel motor programmes may underlie the improvements after skilled-task training, which repetitive movement alone is insufficient to produce (Wolpert et al. 2011).

### Short-interval intracortical facilitation with skill acquisition

To determine if motor skill acquisition is mediated by the same circuits that generate I-waves (Hamada et al. 2014; Sasaki et al. 2018), the present study probed I-wave facilitation using paired-pulse TMS. SICF reflects temporal summation by S2, presumably from activation of intracortical interneuron elements that had been subliminally depolarised by the preceding S1 (Hanajima et al. 2002). Since SICF peaks occur at I-wave periodicities, the interplay between S1 and S2 likely represents the temporal summation of I-waves elicited by the two magnetic stimuli (Hanajima et al. 2002). Robust MEP facilitation was demonstrated at I-wave (~ 1.5 ms) intervals, corroborating previous studies (Ziemann et al. 1998; Di Lazzaro et al. 1999; Hanajima et al. 2002; Peurala et al. 2008) (Table 1). However, at the start of the first session SICF investigated over a range of ISIs showed MEP facilitation at the Peak 1 and 2 interval but not Peak 3 (Table 1). A higher threshold for I3-wave activation may have contributed to this finding (Nakamura et al. 1996; Di Lazzaro et al. 1998b). Epidural recordings have revealed smaller I3-waves than I2-waves (Di Lazzaro et al. 1998a), and TMS studies have reported a smaller MEP amplitude of the third peak than earlier peaks (Ziemann et al. 1998; Cirillo and Perez 2015). These findings indicate that the I3-wave is physiologically distinct from the earlier I-waves and that it is more difficult to recruit than earlier I-waves at the same stimulation intensities.

The present study demonstrated modulation of late I-waves but not early I-waves after skilled training. This finding was consistent with the characteristic modulations of later I-waves reported in other non-invasive brain stimulation techniques (Tokimura et al. 2000; Di Lazzaro et al. 2008, 2009; Lang et al. 2011; Niemann et al. 2018). However, motor training resulted in a reduction of SICF. While these findings are somewhat in line with those from a recent TMS study using the sequential visual isometric pinch task (SVIPT) paradigm that demonstrated a reduction in intracortical facilitation (Mooney et al. 2021), it is unclear what may have given rise to these results. The importance of late I-waves for alpha-motoneuron recruitment with TMS has also been recognised (Thickbroom 2011). Further, selective I-wave modulation is apparent in behavioural contexts, such as visuomotor learning (Hamada et al. 2014) and movement preparation (Cattaneo et al. 2005; Hannah et al. 2018).

### Site of I-wave generation

It is widely accepted that inputs to I-waves arise, at least in part, within M1. Several lines of evidence indicate the involvement of networks outside of M1. For example, following cervicomedullary electrical stimulation, which does not activate intracortical circuits, there was facilitation at I2- and I3-wave peaks (Cirillo and Perez 2015). Additionally, spinal cord injury patients exhibited impaired TMS-induced late I-waves (Cirillo et al. 2016). Based on these findings, subcortical inputs may influence later I-waves. During preparation of visually guided movement, MEP amplitude was facilitated at I2-wave intervals (Cattaneo et al. 2005). The ventral premotor cortex, which is activated during visually guided movements, exerts powerful influence over late I-waves in M1 presumably via long-range cortico-cortical projections (Dum and Strick 1991, 2005; Grezes et al. 2003; Shimazu et al. 2004). These findings indicate that late I-waves may be influenced by elevated activity in cortico-cortical (for example, premotor–motor) projections. Together, modulation of late I-waves may reflect the influence of inputs outside of M1, such as cortico-cortical or subcortical projections.

The mechanism underlying I-wave generation remains a matter of continuous debate. I-waves are abolished under cortical ablation and anaesthesia (Patton and Amassian 1954; Amassian et al. 1987), and reduced by muscimol (Shimazu et al. 2004), indicating that I-waves require excitable and intact grey matter. While the I1-wave may arise from input to the soma of the pyramidal neuron via less complex interneuronal networks, later I-waves may arise from input to the dendrites via more complex interneuronal networks (Di Lazzaro et al. 1998b, 2012). Furthermore, the I1-wave alone may constitute of an earlier phase originating from infragranular layer V and a later phase originating from supragranular layers II/III (Kurz et al. 2019). Evidence also indicates that a spinal contribution to TMS-induced I-waves cannot be excluded (Cirillo and Perez 2015; Cirillo et al. 2016). A full discussion of models for I-wave generation is beyond the scope of the present study (see (Ziemann 2020)). Briefly, the most popular model proposes that corticospinal neurons are repetitively bombarded by physiologically distinct interneuronal subpopulations. The findings of the present study support the view that early and late I-waves may be modulated by physiologically different circuitries within M1. Baseline results revealed different degrees of facilitation at each SICF peak, which indicate that the threshold at which I-waves are recruited may differ between peaks. Indeed, TMS studies and epidural recordings revealed relatively higher activation thresholds of later I-waves (Nakamura et al. 1996; Di Lazzaro et al. 1998a; Cirillo and Perez 2015). TMS studies also showed that early and later onset I-waves are preferentially recruited by PA- and AP-induced currents, respectively (Day et al. 1989; Cirillo and Byblow 2016). Furthermore, learning-induced modulations in late I-waves corroborate findings from non-invasive brain stimulation methods (Di Lazzaro et al. 2008; Lang et al. 2011), behavioural studies (Cattaneo et al. 2005), and pharmacological manipulation (Shimazu et al. 2004). The number of synapses may be one factor mediating the difference between early and late I-wave modulation. The polysynaptic nature of the late I-wave may render it more susceptible to influence by intracortical (Cirillo and Byblow 2016), subcortical (Cirillo and Perez 2015), or cortico-cortical inputs (Shimazu et al. 2004). The results of the present study highlight differences in SICF between early and late I-waves, and as such lend support to the model where inputs from physiologically distinct interneuronal networks produce periodic, repetitive discharge in corticospinal neurons.

### Corticomotor excitability and short-interval intracortical inhibition

Corticomotor excitability was not modulated during and immediately after skill acquisition in both PA- and AP-sensitive circuits. This finding contradicted previous reports of increased corticomotor excitability following motor practise (Coxon et al. 2014; Mooney et al. 2019; Cirillo et al. 2020), whereas others reported no change in excitability with variations of the SVIPT (Paparella et al. 2020) and visuomotor tracking (Smyth et al. 2010). Indeed, there are several reasons why a one-to-one mapping between MEP amplitude and motor output may not be observed during motor learning (Bestmann and Krakauer 2015). In the present study, the lack of modulation of corticomotor excitability could also have been related to the training parameters. A previous study demonstrated an association between modulation of M1 excitability and ballistic pinch, but not ramp pinch (Muellbacher et al. 2001). The present study required participants to execute an intermediate between ballistic movements and fine motor control due to the speed and accuracy demands in the task, which may have influenced the changes to corticomotor excitability commonly observed in purely ballistic movements. Perhaps, a dose effect of training also contributed to the lack of corticomotor modulation (Coxon et al. 2014; Mooney et al. 2019; Cirillo et al. 2020). Also, contrary to our hypothesis, there was no modulation of SICI following motor skill acquisition. Compared to conventional methods, adaptive threshold-hunting using AP-induced current offers a more robust method of probing SICI than PA-induced current by ameliorating confounds (Cirillo and Byblow 2016). Furthermore, adaptive threshold-hunting in AP stimulation is optimal for detecting changes in SICI in a SVIPT paradigm (Cirillo and Byblow 2016). A reduction in GABAergic inhibition following motor learning had been previously reported in TMS (Liepert et al. 1998; Perez et al. 2004; Coxon et al. 2006; Smyth et al. 2010; Mooney et al. 2019), pharmacological (Bütefisch et al. 2000), and magnetic resonance spectroscopy (Floyer-Lea et al. 2006) studies. Learning-induced disinhibition is often associated with improvements in motor performance and supports the role of the GABA_A_ inhibitory system in driving M1 plasticity during skill acquisition. Indeed, SICI disinhibition had been shown to facilitate cortical reorganisation and enhance LTP-like processes (Perez et al. 2004; Coxon et al. 2014), although learning-induced disinhibition is not always a consistent finding. Other reports had found increases (Cirillo et al. 2020) or no significant changes (Rogasch et al. 2009; Cirillo et al. 2010; Berghuis et al. 2017) to SICI after motor skill acquisition. Discrepancies in SICI findings may be related to subtle differences in methodology and the roles of inhibitory mechanisms underlying skill improvements in the present study are inconclusive.

### Limitation and future directions

A limitation of the present study was that the neurophysiological assessments were performed at rest. While the resting condition may not be optimal to reflect task-specific learning adaptations, our parameters cannot be reliably undertaken during task performance. A previous study has shown that the second and third SICF peaks disappear during voluntary contraction (Ziemann et al. 1998). In addition, prolonged experimental sessions may lead to increased participant fatigue and discomfort. For conventional TMS methods, a larger number of trials may be required to reduce MEP variability (Chang et al. 2016; Goldsworthy et al. 2016). However, excellent within-session MEP reliability can be achieved with as low as 5 stimuli (Cavaleri et al. 2017).

One technical limitation of adaptive threshold-hunting using TMS with AP orientation was that 100% maximum stimulatory output was insufficient to elicit THT_adj_ in some participants. As a result, there was a smaller sample for SICI in this study. Compared to conventional SICI, adaptive threshold-hunting using AP stimulation has the benefit of being more robust and sensitive to detect modulations in SICI (Cirillo and Byblow 2016; Cirillo et al. 2018, 2020). Furthermore, threshold-hunting can be used to obtain estimates of SICI more quickly and reliably than conventional methods, which may reduce the required sample size (Samusyte et al. 2018).

Another potential limitation is that interference between SICF and SICI may confound the SICI-readout (Peurala et al. 2008). This confound was unlikely in the present study as the conditioning stimulus intensity fell within the optimal range for maximising SICI (Cirillo and Byblow 2016). SICI was measured at rest using the adaptive threshold-hunting protocol to control for background activity, although future studies may investigate the task-specific changes to SICI.

In the present study, precision, sequence, or a combination of both were likely to contribute to the effects on SICF in the skilled task. Whereas the precision component mainly implicates motor control, the sequential component largely involves cognitive demand. Future studies may investigate the titration of SICF by retaining sequence in the non-skilled task and isolating the effects of precision. The present study was unable to directly investigate the interplay between SICF and SICI using triple stimulation paradigm to better index activity in overlapping neuronal populations.

Since neurophysiological changes were probed within a single session, the present results are constrained to online skill learning only, and potential offline effects were not measured. Skill improvements may continue during consolidation and long-term retention, and future investigations may be directed to learning-induced cortical changes during these phases of motor learning which will likely engage in a wider range of networks outside M1 (Baraduc et al. 2004).

Finally, it is possible that I-wave circuitries are not recruited identically across all populations. Age-related studies had revealed that motor deficits were associated with changes to the characteristics of SICF peaks, with older adults displaying broadened and delayed peaks compared with younger adults (Opie et al. 2018, 2020). TMS-induced late SICF peaks were also impaired in patients with spinal cord injury (Cirillo et al. 2016). Hence, future studies may investigate skill acquisition-induced changes in M1 intracortical circuits in other populations.

## Conclusion

In summary, improvements in skilled performance were successfully acquired by young, neurologically healthy adults who practised a sequential visuomotor finger abduction task. Late SICF peaks were modulated in the skilled task but not in the non-skilled task. Corticomotor excitability and GABA_A_ receptor-mediated inhibition were unchanged, and there were no statistically robust associations between behavioural and neurophysiological measures. While excitatory M1 circuitries responsible for the generation of late I-waves are modulated in the context of motor skill acquisition, their relevance remains unclear.

## Supplementary Information

Below is the link to the electronic supplementary material.

Supplementary file1 (DOCX 545 KB)

## Abbreviations

AMT: Active motor threshold
AP: Anterior–posterior
EMG: Electromyography
FDI: First dorsal interosseous
GABA: Gamma-aminobutyric acid
ISI: Interstimulus interval
LTP: Long-term potentiation
M1: Primary motor cortex
MEP: Motor-evoked potential
PA: Posterior-anterior
PEST: Parameter estimation by sequential testing
RMS: Root mean square
RMT: Resting motor threshold
S1: First stimulus
S2: Second stimulus
SAF: Speed-accuracy function
SICF: Short-interval intracortical facilitation
SICI: Short-interval intracortical inhibition
SVIPT: Sequential visual isometric pinch task
THT: Threshold-hunting target
TMS: Transcranial magnetic stimulation
