# Supplementary material for: Intracortical facilitation and inhibition in human primary motor cortex during motor skill acquisition
Source: Exp Brain Res. 2022 Oct 29;240(12):3289–304. doi: 10.1007/s00221-022-06496-3 (PMC9678989; doi:10.1007/s00221-022-06496-3)

**Supplemental Figures**

**Figure 1.** Correlations between the changes in skill and training performance for skilled and non-skilled task training. Each datum point for each colour represents one participant. N = 18.

**Figure 2**. Correlations between the changes in short-interval intracortical facilitation (SICF) and skill for skilled and non-skilled task training. Left panels, facilitatory peak 1; central panels, facilitatory peak 2; right panels, facilitatory peak 3; top panels, change in SICF from before training to mid training; bottom panels, change in SICF from before training to after training. Each datum point for each colour represents one participant. N = 15.

**Figure 3.** **A.** Short-interval intracortical inhibition (SICI), and **B.** THT; before, mid, and after skilled and non-skilled task training (N = 8). Boxes, 25th and 75th percentiles; whiskers, 10th and 90th percentiles.

**Figure 4**. Correlations between the changes in short-interval intracortical inhibition (SICI) and skill for skilled and non-skilled task training. Left panel, change in SICI from before training to mid training; right panel, change in SICI from before training to after training. Each datum point for each colour represents one participant. N = 8.

**Supplemental Figures**

**Figure 1**


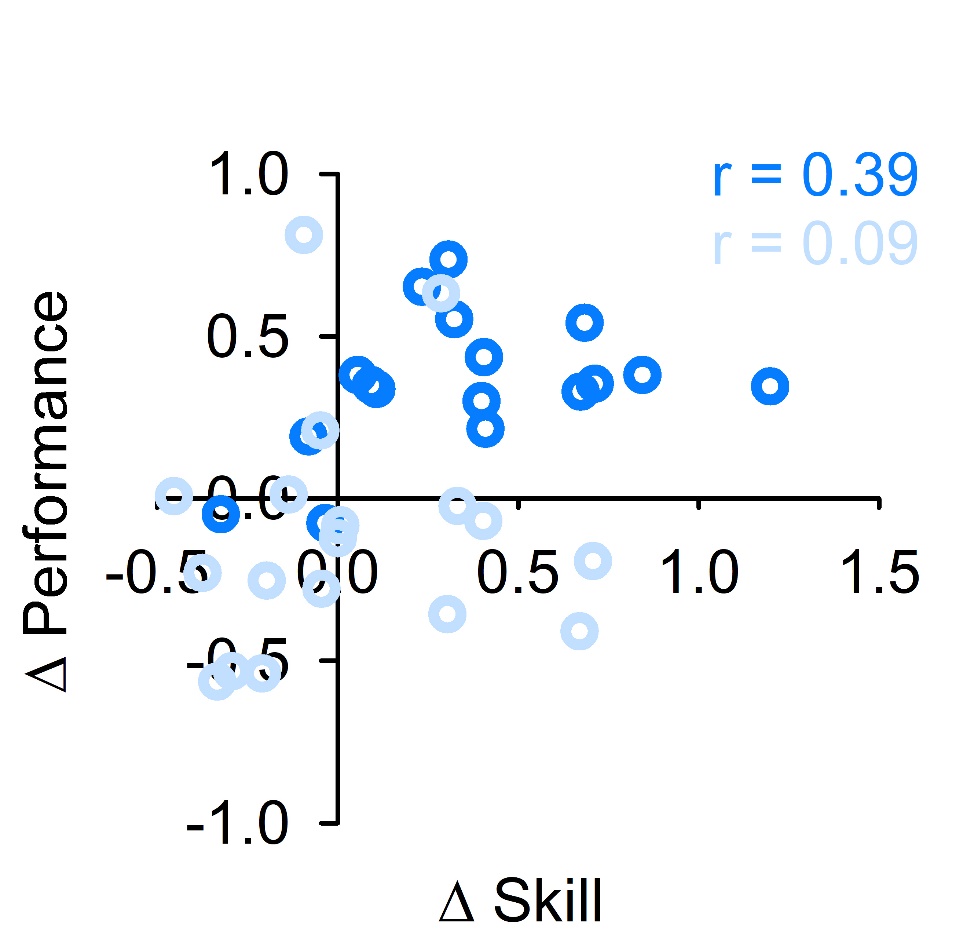


**Figure 2**


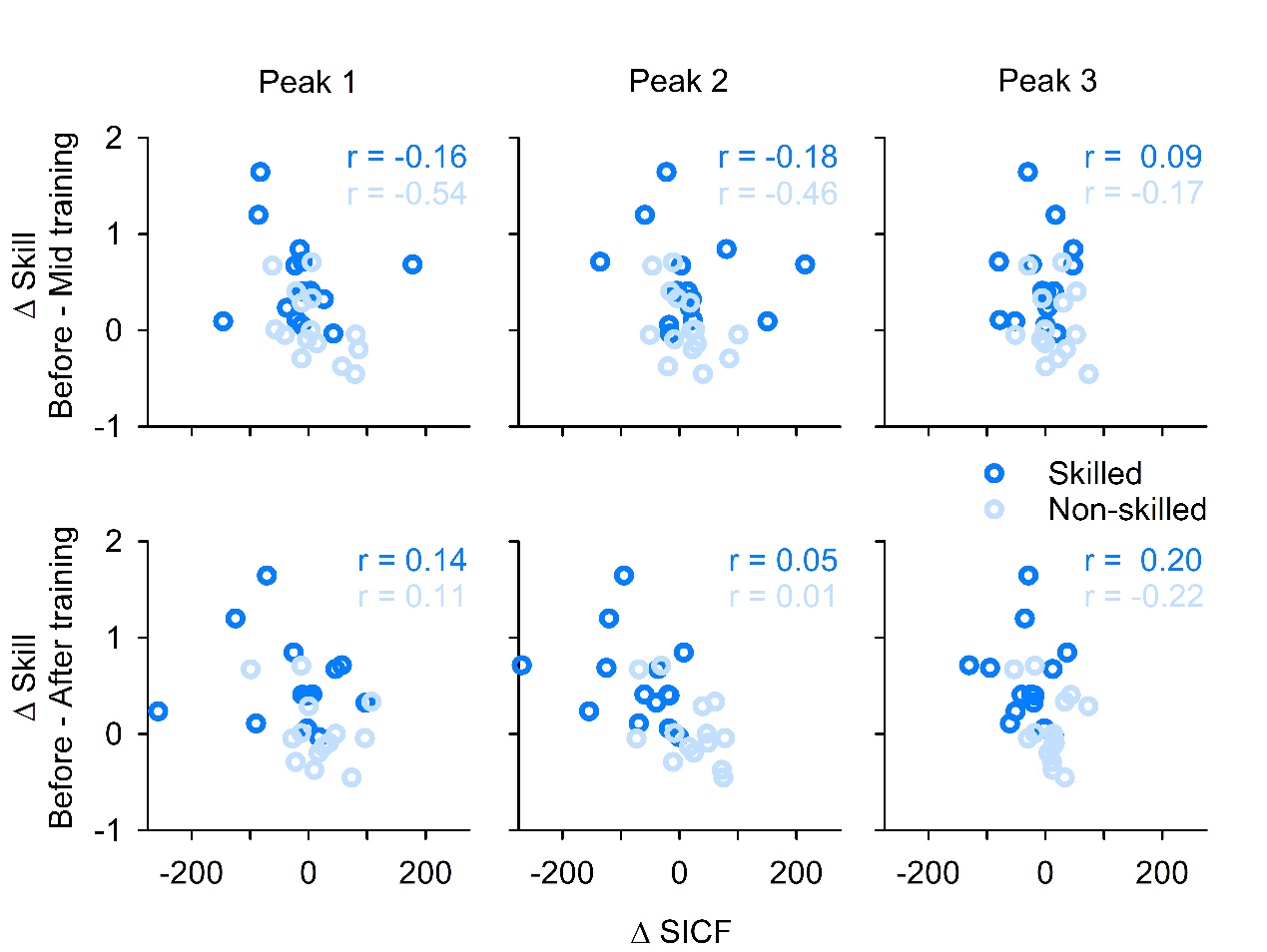


**Figure 3**


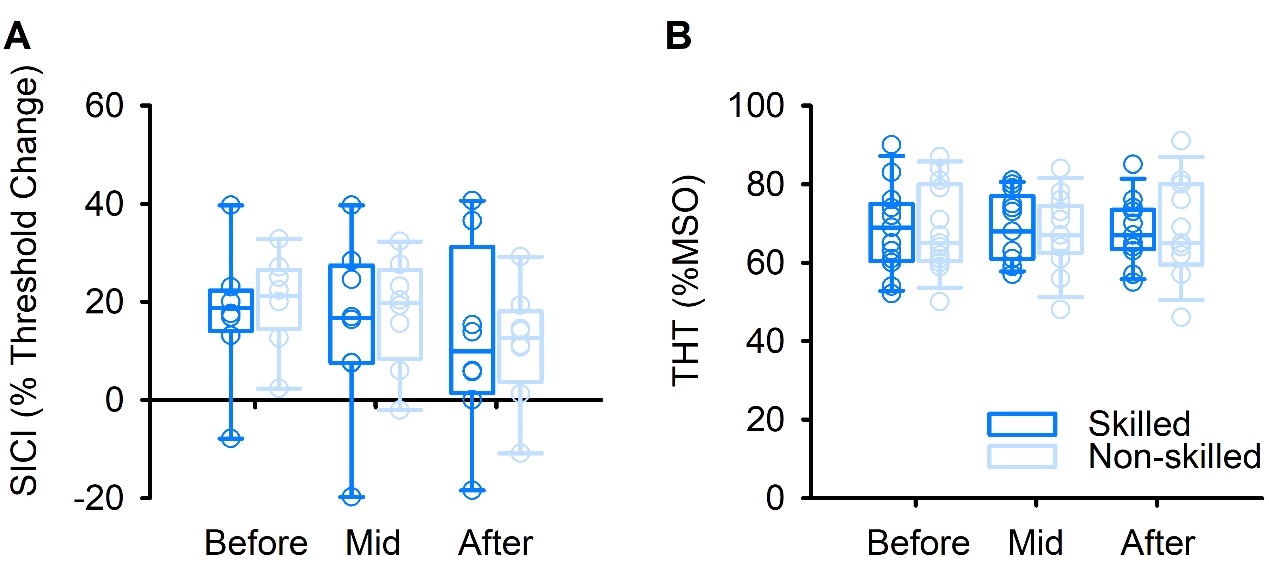


**Figure 4**


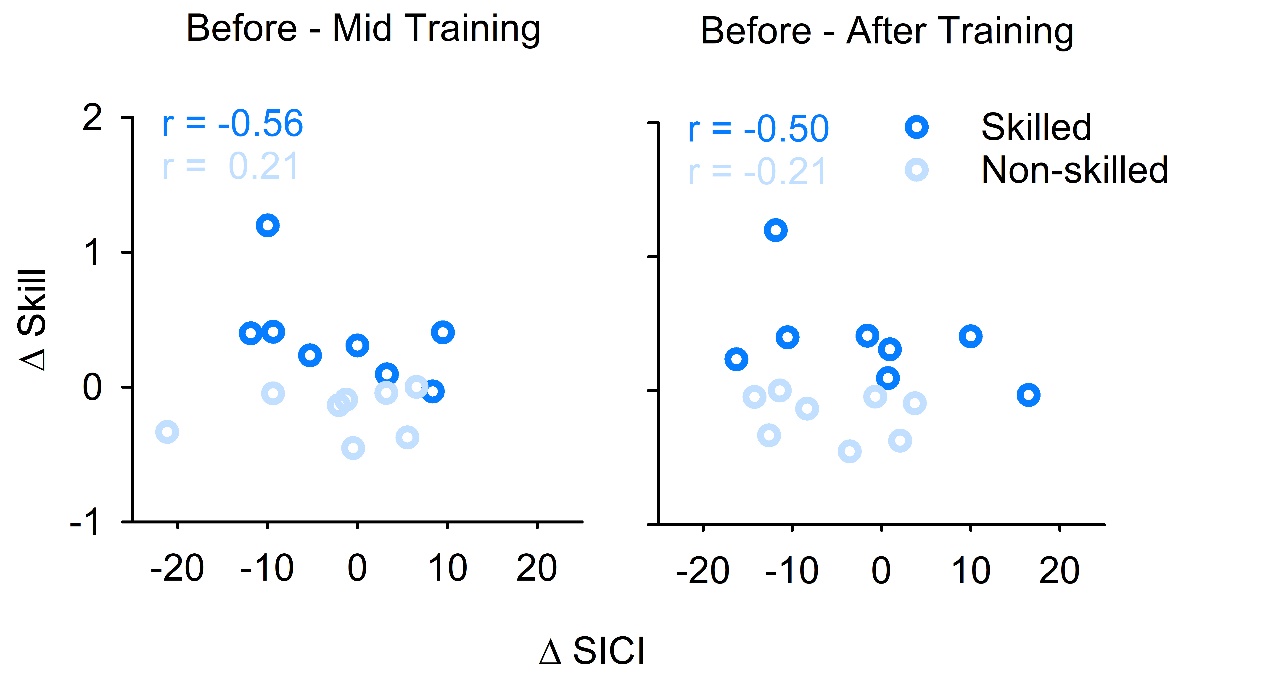

Supplement: Supplementary file 1 — Supplementary file1 (DOCX 545 KB) [file 221_2022_6496_MOESM1_ESM.docx]
